# A Novel Vehicle Detection Method Based on the Fusion of Radio Received Signal Strength and Geomagnetism

**DOI:** 10.3390/s19010058

**Published:** 2018-12-24

**Authors:** Liangliang Lou, Jinyi Zhang, Yong Xiong, Yanliang Jin

**Affiliations:** 1 Key laboratory of Specialty Fiber Optics and Optical Access Networks, Joint International Research Laboratory of Specialty Fiber Optics and Advanced Communication, Shanghai Institute for Advanced Communication and Data Science, School of Communication and Information Engineering, Shanghai University, Shanghai 200072, China; liangliang.lou@mail.sim.ac.cn (L.L.); zhangjinyi@staff.shu.edu.cn (J.Z.); wuhaide@shu.edu.cn (Y.J.); 2 Shanghai Institute of Microsystem and Information Technology, Chinese Academy of Science, Shanghai 200050, China

**Keywords:** vehicle detection, geomagnetism, received signal strength, two-sensors fusion

## Abstract

A geomagnetic signal blind zone exists between the front and rear axle of high-chassis vehicle such as trucks and buses, which leads to multiple-detection problem in detecting those vehicles running at low speed on roads or error-detection problem in the case of the stopping position of the vehicle is not standard when waiting for the traffic light to change. In order to improve the detection accuracy of any type of vehicle running at any speed, a novel two-sensors data fusion vehicle detection method through combining received signal strength from radio stations with geomagnetism around vehicles is designed and verified in the paper. Experimental results show that the accuracy of our proposed method can reach 95.4% and traditional single magnetism-based detection method was only 83.4% in the detection of high-chassis vehicles.

## 1. Introduction

Nowadays, a lot of cities have started to look for intelligent transportation systems. The main principle of intelligent transportation systems is to assign the appropriate traffic signal time or manage the parking lots intelligently according to the information collected by vehicle detectors [1].

At present, there are various vehicle detection methods, such as inductance loop [2,3,4], geomagnetic sensor [5], microwave radar, infrared, video, etc. The video vehicle detector has the advantages of high detection accuracy, stability and reliability and widely used in the world. It not only can be used to monitor road conditions, capture speeds, count vehicles, classify vehicle also plays an important role in the reduction of the number of road accidents [6]. However, the cost of video vehicle detectors is still relatively high compared with other vehicle detectors and many traffic information collection scenarios do not require image data. 

The inductance loop vehicle detector is widely used because of its low cost, strong practicability and high accuracy. However, it is very difficult to install and maintain the inductance loop vehicle detectors on the roads, the surface structure of the roads will be seriously damaged and the aesthetics of the roads or parking spaces will be seriously affected by the installed inductance loop vehicle detectors. Due to the power supply problem, it is difficult to install the inductance loop vehicle detectors in some scenarios such as the roadside parking space monitoring application.

Generally, the battery-powered geomagnetic sensor vehicle detectors are available 5–10 cm in height and 5–15 cm in diameter. And the cost of those detector is about $35 in China and the working life of those is up to 3 years. Therefore, the geomagnetic sensor vehicle detector has the advantage of low power consumption, low cost and easy deployment. The geomagnetic sensor vehicle detectors have been widely used for collecting traffic data and gradually replaced the inductance loop vehicle detectors in a variety of occasions in recent years.

The earth is a natural magnet with a surface magnetic field of about 500–600 mGs. The magnetic field remains relatively stable in a certain area. When a ferromagnetic substance such as vehicle having a magnetic permeability is present in the area, the magnetic field of the region is disturbed, resulting in the magnetic strength becoming larger or smaller [7]. Geomagnetic sensor is a kind of chip that can convert magnetic signal into electrical signal. Therefore, vehicles can be detected by analyzing the change in the magnetic field around the geomagnetic sensor.

However, there is a geomagnetic signal blind zone between the front and rear axle of a vehicle [8]. The blind zone is very obvious in the high-chassis large vehicles such as trucks, buses, SUVs (Sport Utility Vehicles), etc., which leads to multiple-detection problems in detecting those vehicles [9] when running at low speed on roads. Simultaneously, the stopping position of a vehicle is not standard when waiting for the traffic light to change or parking on a parking lot, the geomagnetic signal blind zone problem will cause the vehicle to be undetected by the vehicle detector with the traditional single magnetism-based method. 

The vehicle actuated signal control strategy has been widely adopted in traffic control system and requires the real-time information about the presence of vehicles from vehicle detectors installed on roads. The inaccurate vehicle detection results will cause the traffic signal controller to assign wrong signal timings when the traffic signal controller equipped with the vehicle actuated signal control strategy [10]. What is more, the coordinated adaptive traffic control system such as Sydney Coordinated Adaptive Traffic System (SCATS), which requires number of spaces and total space time during green of each cycle to implement maximum throughput, minimum stops and minimum delay strategies [11]. The multiple-detection problem will cause the number of spaces and total space time to be incorrectly calculated and the coordinated adaptive traffic control strategy of the SCATS will be affected.

Simultaneously, the speed of vehicles entering and leaving the parking lot are generally less than 20 km/h, the multiple-detection problem will lead to large statistical errors. The error-detection of the parking spaces’ status will bring certain difficulties to the managers of the parking lot. What is more, the detection result of most vehicle detectors is transmitted through the wireless channels, the wrong detection result will increase the transmission times which resulting in much higher power consumption of the vehicle detector and probability of wireless channel collision with the other vehicle detectors. This problem is much more obvious on the industrial district roads and suburban roads where large vehicles account for a larger proportion of total traffic flows.

In order to solve the multiple-detection problem in detecting those vehicles running at low speed on roads or error-detection problem in the case of the stopping position of the vehicle is not standard when waiting for the traffic light to change caused by the blind zone of geomagnetic signals in vehicle detection procedure, the multi-sensor data fusion detection methods of combining with optical sensor [12] and acceleration sensor [13] were proposed, experiments results showed that those methods did improve vehicle detection accuracy. However, the optical sensor can be easily affected by environmental conditions, such as dust, rain and fog, etc., which leads to misjudgment of the state of vehicles. The vibration signal generated by vehicles is so weak that an expensive high-resolution acceleration sensor was required.

The electromagnetic waves exist widely and always affect our lives. A novel radio-based vehicle detection method was proposed in the literature [14]. A lot of Wi-Fi APs (Access Point) were deployed on a roadside by the authors of the literature. And the vehicle detection and speed estimation methods are realized by analyzing the change in received signal strength of Wi-Fi signal collected by the laptops placed on the other roadside. The literature has provided a new way for vehicle detection methods.

In order to improve the detection accuracy in detecting long wheelbase and high chassis large vehicles running at low speed or stopping at non-standard positions when waiting for the traffic light to change on a road or parking on a parking lot, we add a FM (Frequency Modulation) radio module in the traditional single magnetism-based vehicle detector, propose a two-sensors data fusion vehicle detection method which is realized through combining the received signal strength collected by the FM radio module and the geomagnetism around vehicles sampled by a geomagnetic sensor. 

We build test scenarios to verify the performance of the proposed method by installed vehicle detectors with the proposed method and traditional single magnetism-based method on the same road. The experiment results show that our proposed two-sensors data fusion vehicle detection method bring higher detection accuracy than the traditional single magnetism-based detection method. 

The method we proposed has the advantages of easy implementation and low cost, which is very suitable for vehicle detection applications. The higher detection accuracy of vehicle detector will lay the foundation for intelligent decision making in intelligent transportation systems.

## 2. Detection System Architecture and Signal Characteristics

### 2.1. System Architecture of Vehicle Detection

**Figure 1:**
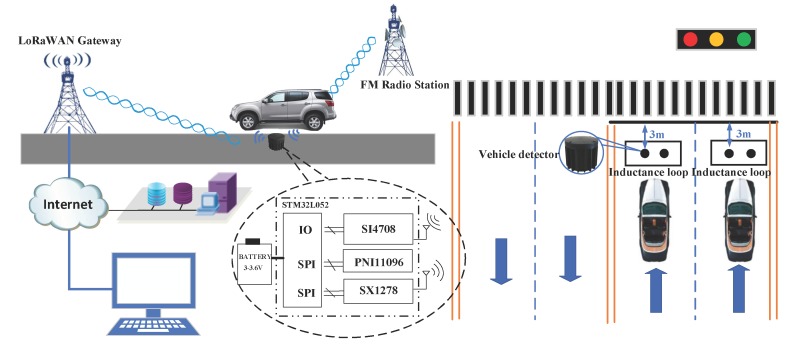
Vehicle detection system architecture and installation diagram.

The vehicle detection system is composed of a LoRaWAN (LoRa Wide Area Network) gateway, vehicle detectors and a cloud server. The vehicle detector is made up of a battery ER34615M produced by Ultralife company, Shenzheng, China, a geomagnetic sensor PNI11096 produced by PNI company, Santa Rosa, CA, USA, a FM radio module SI4708 produced by Silicon Labs company, Austin, TX, USA, a LoRa module SX1278 produced by Semtech company, Camarillo, CA, USA, and a microcontroller STM32L052 produced by STMicroelectronics company, Geneva, Switzerland. The detection system architecture and installation diagram are shown in Figure 1.

The PNI11096 is a low cost magnetic measurement chip produced by U.S. PNI company and connects with the microcontroller through a SPI interface in our circuit board. The chip can control and measure three independent magneto-inductive sensors and each sensor is individually selectable for measurement. The power consumption is only 1 μA at idle mode and 1.5 mA at sampling mode [15]. Therefore, the chip is very suitable for the power-constrained wireless vehicle detectors.

The SI4708 is a tiny FM radio receiver chip produced by Silicon Labs company, Austin, TX, USA. It requires only 6.25 mm^2^ of board space and a minimum component count. The SI4708 has highly flexible functionality catering to the subjective nature of audio preferences and variable FM broadcast environments worldwide. Two-wire slave-transceiver and three-wire interfaces are provided for the microcontroller to read and write the control registers of the chip. The RSSI (Received Signal Strength Indicator) level will be available by reading from the bits RSSI [7:0] of register 0AH when the seek operation completes [16]. 

The SX1278 transceivers feature the long range modem that provides ultra-long range spread spectrum communication and high interference immunity whilst minimizing current consumption. The chip can achieve a sensitivity of over −148 dBm using a low cost crystal and bill of materials. The high sensitivity combined with the integrated +20 dBm power amplifier yields industry leading link budget making it optimal for any application requiring range or robustness [17]. The LoRa modulation technique also provides significant advantages in both blocking and selectivity over conventional modulation techniques, solving the traditional design compromise between range, interference immunity and energy consumption. Therefore, the chip is widely used in IoT (Internet of things) devices. In our application, it is programed by the microcontroller STM32L052 through SPI (Serial Peripheral Interface) interface. 

The ultra-low-power STM32L052 microcontroller with the high-performance ARM Cortex-M0+ 32-bit RISC core and the maximum operating frequency can up to 32 MHz [18]. The microcontroller not only undertakes the collection of geomagnetic signals and electromagnetic wave signal strength also undertakes the analysis and processing of these data to form the vehicle detection results and transmits these results to the LoRaWAN gateway through SX1278 chip. 

The LoRaWAN gateway, as a core device, is installed within the effective communication distance of the vehicle detectors. The LoRaWAN gateway will transmit the collected vehicle detection results to the cloud server through the Internet. Finally, the results will be displayed on various terminals.

### 2.2. Geomagnetic Signal Characteristics Analysis

**Figure 2:**
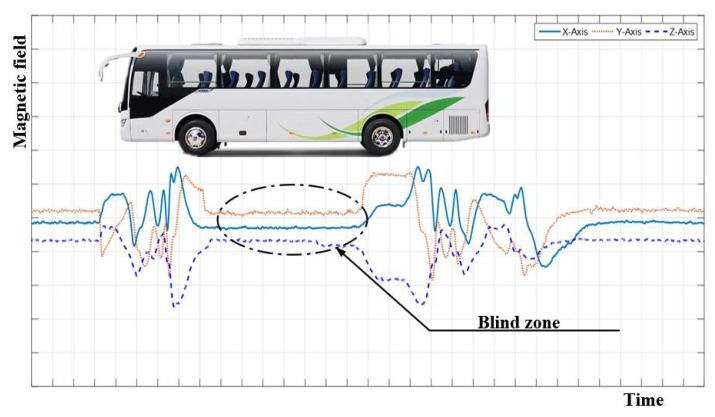
The magnetic signal blind zone problem of high chassis vehicles.

The geomagnetic disturbance signal caused by a vehicle is shown in Figure 2. From Figure 2, we can see that the area of front and rear wheels has the strongest magnetic disturbance signal. However, there is a magnetic signal blind zone between the front and rear wheel, which may cause misjudgment of vehicle status.

### 2.3. Received Signal Strength Characteristics Analysis

Electromagnetic wave propagation loss in free space according to the Friis formula is:

(1) Pr=PtDtDr(λ4πR)2,

where *P_r_* is the received power, *P_t_* is the transmit power, *D_t_* is the gain of the transmit antenna, *D_r_* is the gain of the receive antenna, *R* is the distance between the receiver and the transmitter and λ is the wavelength of electromagnetic wave. 

**Figure 3:**
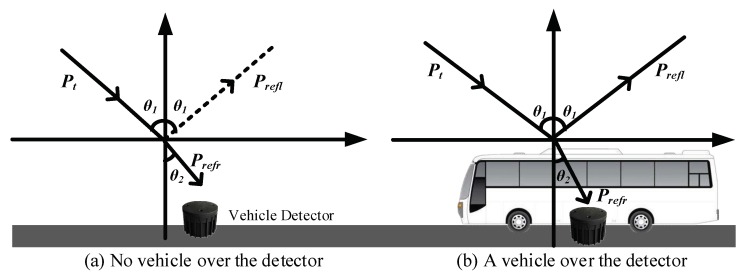
Simplified model of radio signal caused by vehicle influence.

Due to electromagnetic waves have reflection, refraction, diffraction and other phenomena during the propagation. However, the doppler effect of electromagnetic waves which will increase reflection while increasing the received signal strength is not obvious when vehicle was running at low speed or static. In order to simplify the model, we only consider the reflection and refraction of electromagnetic waves, the simplified model is shown in Figure 3.

According to the law of conservation of energy, we can get the following formula:

(2) $$P_{t}=P_{refl}+P_{refr},$$

where *P_refl_* is the electromagnetic wave energy reflected by the vehicle. *P_refr_* is the electromagnetic wave energy refracted by the vehicle. Substituting Equation (2) into Equation (1) we can get:

(3) $$P_{r}=\left(P_{refl}+P_{refr}\right)D_{t}D_{r}{\left(\frac{\lambda }{4\pi R}\right)}^{2},$$

In Equation (3), the two parameters of *R* and *λ* remain unchanged. From Figure 3a, we assume that the receiver can fully receive the electromagnetic wave energy from the transmitter when no vehicle is present on the receiving antenna. In other words, the electromagnetic wave energy is not reflected by the vehicle. If there is a vehicle on the receiving antenna, the vehicle will reflect and refract the electromagnetic waves, resulting in lower electromagnetic energy received by receiver than no vehicle above it. Therefore, when a vehicle enters the sensing area of the detector, the received signal strength (RSS) value sampled by the receiver will be changed significantly. 

## 3. Detection Method Design and Implementation

Before designing the two-sensors data fusion vehicle detection method, we first analyze the data characteristics of geomagnetism and received electromagnetic wave’s signal strength. We built a test-bed to collect and analyze the geomagnetism and received signal strength data. 

Generally, the speed of vehicles is 5–80 km/h on urban roads, the length of vehicles is 3–5 m and the distance between the front and rear vehicles on the same lane is 2–30 m. From the above traffic parameters, we can see that the vehicle’s retention time above the sensor is generally more than 200 ms and the interval between the front and rear vehicles is generally more than 1 s. According to the Nyquist Theorem, as long as the sampling rate of the sensor is greater than 10 Hz, the vehicle running on the urban roads can be detected accurately. 

In order to obtain as many characteristics of sensor signals as possible, we use a geomagnetic sensor to collect the *Z*-axis of the geomagnetic signals and the sampling rate is 100 Hz. The received signal strength data collected by a FM radio module and the sample rate is also 100 Hz.

The verification circuit board of the test-bed is placed on the middle of a road. A USB (Universal Serial Bus) to UART (Universal Asynchronous Receiver/Transmitter) serial cable is used to connect the microcontroller UART interface to a USB port of the laptop placed on the roadside and the baud rate of the UART interface is 115,200 bps. Microcontroller will start the geomagnetic sensor to sample the geomagnetic signal and the FM module to estimate the received signal strength at the frequency of 100 Hz which is generated by a timer of the microcontroller. After that, the microcontroller will enter into sleep mode. Once the microcontroller is woken up by the periodic timeout event, it will read the magnetic data from the geomagnetic sensor and the received signal strength data from the FM radio module. Then, the collected geomagnetic data and the received signal strength data will be sent by the microcontroller to laptop for further analysis. 

**Figure 4:**
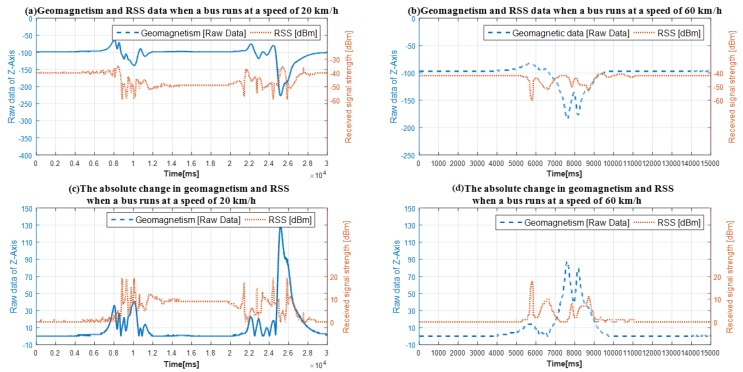
The geomagnetism and RSS data when a bus runs at different speeds.

In order to collect and analyze the characteristics of geomagnetic signal and received signal strength disturbed by the high-chassis vehicle, we use a bus with the length of 11 m to pass over the sensors at different speeds, the speeds are 10 km/h and 60 km/h. The collected data shown in Figure 4.

In Figure 4, the geomagnetic data is raw data read from the corresponding register of geomagnetic sensor, so there is no unit of the measurement. The received signal strength is the value from the corresponding register of the FM radio module and calculated according to the module’s datasheet. Therefore, the unit of the received signal strength is dBm. We also analyze the absolute changes in geomagnetic signal and received signal strength as shown in Figure 4c,d.

From Figure 4a,c we can see that the area between point 7500 and 12,500 of *x*-axis is the data of geomagnetism and received signal strength when the front wheel of the bus was passing. The area between point 12,500 and 20,000 of *x*-axis is the data of the vehicle body between the front and rear wheel. The area between data point 20,000 and 27,500 of *x*-axis is the sensors’ data when the rear wheel of the bus was passing.

From Figure 4a,c we can also see that there is an obvious geomagnetic signal blind area between data point 12,500 and 20,000 of *x*-axis. Fortunately, we find that the received signal strength data has obvious signal characteristics in the geomagnetic signal blind area. This phenomenon provides the possibility to realize two-sensors data fusion detection method in a vehicle detector, which can improve the detection accuracy of large vehicles running at low speed.

We also analyzed the characteristic of the geomagnetic signal and received signal strength data when the bus running at high speed. From Figure 4b,d we can find that the geomagnetic signal blind zone is not as obvious as the bus running at low speed and the received signal strength also shifts drastically as same as geomagnetic signal when the vehicle was passing.

Therefore, if the data of geomagnetism and received signal strength are effectively fused in a vehicle detection method, it will improve the detection accuracy of different types of vehicles running at various speed. 

### 3.1. Adaptive Adjustment Algorithm for Reference Baselines

According to the analysis above, if the amount of change in geomagnetism and received signal strength is greater than decision thresholds, it can be considered a vehicle is detected [19,20]. However, the geomagnetism and received signal strength are easily affected by environmental factors such as temperature and humidity, which cause the reference baselines to drift and affect the vehicle detection accuracy. In this paper, we use the following weighted average method to update the reference baselines:

(4) $$B\left(n\right)=\{\begin{array}{c} B\left(n-1\right)*\left(1-\alpha \right)+\alpha *A\left(n\right)\;\left(n>0\;and\;no\;vehicle\;exists\right)\;\\ B\left(n-1\right)\;\left(n>0\;and\;vehicle\;exists\right)\;,\;\\ A\left(n\right)\;\left(n=0\right)\; \end{array}$$

where *n* is the serial number of sampling point. Where *α* is a weighting coefficient and the value of *α* is 0.05 in the paper. *B*(*n*) and *B*(*n*−1) is the current and previous reference baseline of geomagnetism or received signal strength. *A*(*n*) is the current sampled data of geomagnetism or received signal strength. From Equation (4) we can see that the first sampled value will be used as the reference baselines. After that, the reference baselines only can be updated when no vehicle is detected. Otherwise, it will remain unchanged. 

### 3.2. Vehicle Detection Algorithm Design

In this paper, the state machine mechanism is used to implement our proposed two-sensors data fusion vehicle detection method. For the sake of easy analysis, we binarized the relevant results before the method is implemented.

(5) $$F\left(n\right)=\{\begin{array}{c} 1,\;abs\left(\;A\left(n\right)-B\left(n\right)\right)\geq T\left(n\right)\;\\ 0,\;otherwise\; \end{array}$$

In Equation (5), *T*(*n*) is the decision threshold of magnetism-based or received-signal-strength-based vehicle detection method, the values are derived from a large amount of data analysis. *F*(*n*) is the binary vehicle detection result of magnetism-based or received-signal-strength-based detection method, a value of 1 indicates that a car has been detected and vice versa. 

The proposed two-sensors data fusion vehicle detection method is achieved by fusing the data of geomagnetism and received signal strength to overcome the limitations and instability of traditional single magnetism-based detection method. The algorithm principle is shown as follows:

(6) $$D\left(n\right)=\{\begin{array}{c} 1,\;if\left(\;D\left(n-1\right)==0\;and\;F_{mag}\left(n\right)==1\;and\;n>0\right)\;\\ 0,\;if(\left(D\left(n-1\right)==1\;and\;F_{mag}\left(n\right)==0\;and\;F_{rss}\left(n\right)==0\;and\;n>0\right)\\ or\;\left(n==0\right))\;\\ unchanged,\;otherwise\; \end{array},$$

where *D*(*n*) and *D*(*n*−1) is the current and previous binarization vehicle detection status of our proposed two-sensors data fusion vehicle detection method. *F_mag_*(*n*) is the current binarization detection result of the magnetism-based detection method according to Equation (5). *F_rss_*(*n*) is the current binarization detection result of the received-signal-strength-based vehicle detection method according to Equation (5). From Equation (6), we can consider a vehicle detected when the amount of change in the geomagnetic signal is greater than decision threshold and left when the amount of change both in the geomagnetic signal and the received signal strength are less than the respective decision threshold.

Due to the existence of geomagnetic signal blind zone, it may cause the value of the binarization result *F_mag_*(*n*) to change between 0 and 1 in short time. To overcome this problem, we introduce a continuous counting mechanism to eliminate the misjudgment caused by the short-term jump, the three introduced counters are vehicle entering counter *CNT_vehicle-entering_*, magnetism-based method vehicle leaving counter *CNT_mag-based-leaving_* and received-signal-based method vehicle leaving counter *CNT_rss-based-leaving_*. Since the sampling rate of sensors are 100 Hz, according to the Nyquist Theorem and general traffic parameters, the vehicle entering counter’s threshold *Cy* is set to 10. 

Generally, if the speeds of vehicles are fast on a road, the distance between the front and rear vehicles on the same lane will be much larger than that running at the low speeds. Therefore, the time interval between the front and rear vehicles running at fast speeds is similar to that running at low speeds. According to the above analysis, the vehicle leaving counter’s threshold *Cn* is set to 30 in the paper. Overall, the vehicle detection status will change only when the value of each counter is greater than the respective threshold.

**Figure 5:**
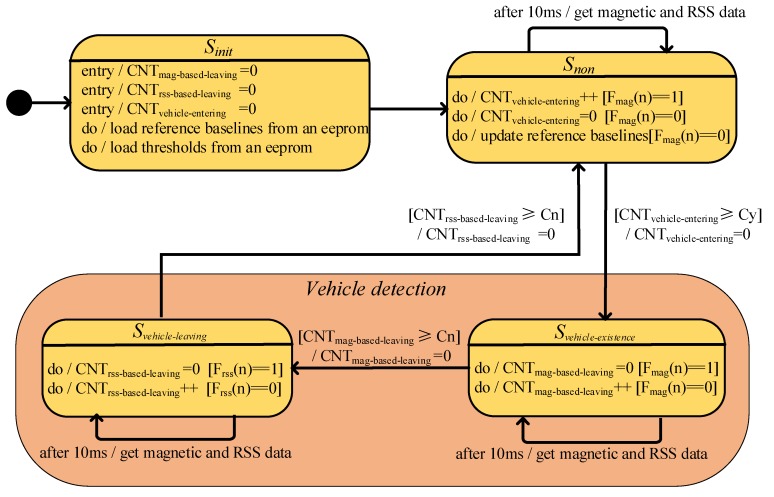
The state machine of the two-sensors data fusion vehicle detection method.

The proposed two-sensors data fusion detection method in the paper is implemented based on the state machine mechanism. The state machine contains 4 states and is realized as shown in Figure 5:

*S_init_* initializes the value of relevant parameters.
*S_non_* measures the length of the high level magnetic pulse and updates reference baselines.
*S_vehicle-existence_* measures the length of the low level magnetic pulse and detects a vehicle.
*S_vehicle-leaving_* measures the length of the low level RSS pulse and waits the vehicle to leave.

## 4. Verification and Analysis

In order to verify the performance of the two-sensors data fusion vehicle detection method proposed in the paper, we installed vehicle detectors with the proposed method on a road. For comparison, we also installed vehicle detectors with the traditional single magnetism-based method and inductance loop vehicle detectors in the same lane. The detectors installation diagram shown in Figure 1. 

The algorithm principle of the traditional single magnetism-based method has been described deeply in the literature [5,19,20]. Generally, the vehicle existence detected based on the change in the magnetism is greater than a specific threshold, the vehicle leaving detection is based on the change in the magnetism is less than a specific threshold within a certain time window. There is no *S_vehicle-leaving_* state in the traditional single magnetism-based vehicle detection method. In other word, if *CNT_mag-based-leaving_* is greater than *Cn*, the state will jump to *S_non_* and end a complete vehicle detection procedure. 

We used two methods to verify the performance of the proposed vehicle detection method: (i) We took the manual statistical results as a reference, compared the different types of vehicle detection accuracy of our proposed two-sensors fusion method and traditional single geomagnetic-based method at different time of a day. (ii)We took inductance loop vehicle detectors’ statistical results as a reference and compared overall detection accuracy of the detectors with traditional single magnetism-based method and the proposed two-sensors data fusion method up to 30 days.

### 4.1. Accuracy Analysis in Setecting Different Vehicle Types at Different Periods

The peak hours of test road are 07:00–09:00 and 17:00–19:00. The average speed of vehicles is about 25 km/h during peak time and 50 km/h during idle time. In order to verify the detection accuracy of different vehicle types at different periods, this paper selects six time periods to verify the accuracy of the traditional single magnetism-based and the proposed two-sensor data fusion vehicle detection method. 

Generally, the length of family car is generally less than 5 m [21] and the most of the vehicles run on the urban roads are family cars. For the sake of statistical simplification, a big vehicle refers to a vehicle with the length greater than 5 m, such as buses, trucks, etc. And a small vehicle refers to a vehicle with the length less than 5 m, such as family cars.

From Table 1, we can find that both the traditional single magnetism-based detection method and our proposed method have higher detection accuracy in the idle time than that in the peak time of the road. Simultaneously, the traditional single magnetism-based method obviously has multiple-detection problems in the detection of large vehicles during the peak period. The main reason is that the average speed of vehicle is slow during peak time or the stopping position of vehicle is not standard when the vehicles waiting for the traffic light, the blind zone problem causes the same vehicle to be detected multiple times by the detector with the traditional single magnetism-based algorithm. 

From Table 1, we can know that the detection accuracy of our proposed two-sensors data fusion detection method has relatively higher precision than the traditional single magnetism-based method in the large vehicle detection process, the detection accuracy of ours is up to 95.4% while traditional singlemagnetism-based method is only 83.4%. However, the detection accuracy of ours is similar to the traditional method in the small vehicle detection process. Therefore, above analysis shows that our proposed method can significantly improve the large vehicles detection accuracy, effectively solve the problem of multiple-detection of those vehicles running at low speeds.

From Table 1, we can also find that whether large or small vehicles, the detection accuracy of the inductance loop vehicle detector is higher than the vehicle detectors with the traditional single magnetism-based detection method and our proposed method. The main reason is that the sensing size of inductive loop vehicle detector is large, resulting in the blind zone is smaller than the vehicle detectors with the traditional single magnetism-based detection method and our proposed method.

### 4.2. Long-Term Stability Verification

In order to verify the long-term stability of the proposed method in the paper, we used the statistical data of the inductance loop vehicle detector as a reference. The experimental data from 1 October to 30 October in 2018 is shown as follows.

**Figure 6:**
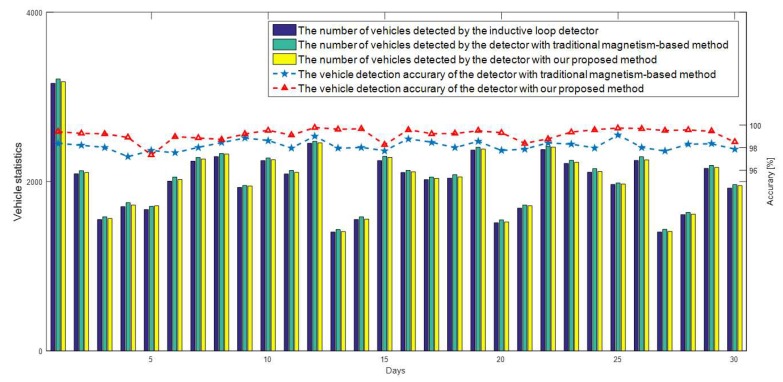
Long-term vehicle statistics and accuracy comparison.

From Figure 6, we can also see that the overall accuracy of our proposed two-sensors fusion method is 0.97% higher than that of the traditional single magnetism-based detection method. The main reason for the result is that most of the large vehicles running on the tested road are public buses and commuter buses, which will be out of service after 7 PM. What is more, there are very few big trucks and pickup trucks running on the tested road and the small vehicles account for a high proportion of the total traffic flow on the tested road. 

From Table 1, we can find that the small vehicle detection accuracy of ours is as similar as that of traditional single magnetism-based detection method. Due to the above several reasons, the overall accuracy of our proposed method is only slightly improved compared with the traditional single magnetism-based vehicle detection method in the long-term test. From the above analysis, we can find that the proposed method can work as stable and reliable as the inductance loop and the traditional single magnetism-based vehicle detectors.

## 5. Conclusions

The paper proposes a two-sensors data fusion method through combining the geomagnetism and the received signal strength, to solve the multiple-detection problem of the traditional single magnetism-based detection method in high-chassis large vehicle detection and error-detection problem in the case of the stopping position of the vehicle is not standard when waiting for the traffic light to change. The proposed method was verified on a road, experiment results show that our designed method has higher detection accuracy compared with traditional singlemagnetism-based detection method.

The proposed method can greatly improve the large vehicle detection accuracy, especially is suitable for the scenarios where large vehicles account for a high proportion of the total traffic flow such as industrial district roads and suburban roads. Simultaneously, the higher detection result will decrease the transmission and re-transmission times which lead to lower power consumption of the wireless transceiver module and smaller probability of channel collision with the other detectors. Moreover, the method we proposed has the advantages of easy implementation and stability as same as the traditional single magnetism-based vehicle detector. 

However, compared with the traditional single magnetism-based detector, the FM radio module in our proposed method not only increases the cost of about $2 also increases the additional power consumption of about 10 mWh at the sampling rate of 100 Hz. In the future, we will pay more attention to the low-power characteristics of the proposed method. For example, the method of the adaptive adjustment of geomagnetic sensor and FM module sampling rate according to the vehicle speed, the mechanism of using geomagnetic signal change to trigger FM module to start working will be studied and implemented in the future work.

## Figures and Tables

**Table 1:** Short-term vehicle detection statistical results.

Periods	Manual Statistic		Single Magnetism-Based Method		Proposed Two-Sensors Data Fusion Method		Inductance Loop Vehicle Detector	
	big vehicle number	small vehicle number	big vehicle number/accuracy	small vehicle number/accuracy	big vehicle number/accuracy	small vehicle number/accuracy	big vehicle number/accuracy	small vehicle number/accuracy
07:30–07:50	21	72	24/85.7%	75/95.8%	22/95.2%	73/98.6%	22/95.2%	72/100%
08:00–08:20	19	81	24/73.6%	83/97.5%	20/94.7%	82/98.7%	19/100%	82/98.7%
12:00–12:20	7	39	8/85.7%	40/97.4%	7/100%	39/100%	7/100%	39/100%
12:30–12:50	10	32	11/90.0%	32/100%	11/90.0%	33/96.8%	10/100%	32/100%
17:00–17:20	28	89	32/85.7%	92/96.6%	29/96.4%	93/95.5%	28/100%	90/98.9%
17:30–17:50	24	85	28/83.3%	85/100%	25/95.8%	86/98.8%	24/100%	85/100%
Total	109	398	127/83.4%	407/98.0%	114/95.4%	406/98.2%	110/99.0%	400/99.4%
